# Early steroid pulse therapy among children with influenza virus-associated encephalopathy

**DOI:** 10.1186/s40560-020-00479-8

**Published:** 2020-08-12

**Authors:** Takeshi Hatachi, Nobuaki Michihata, Muneyuki Takeuchi, Hiroki Matsui, Kiyohide Fushimi, Hideo Yasunaga

**Affiliations:** 1 Department of Intensive Care Medicine, Osaka Women’s and Children’s Hospital, 840 Murodocho, Osaka, Izumi 594-1101 Japan; 2 Department of Health Services Research, Graduate School of Medicine, The University of Tokyo, Tokyo, Japan; 3 Department of Clinical Epidemiology and Health Economics, School of Public Health, The University of Tokyo, Tokyo, Japan; 4 Department of Health Policy and Informatics, Tokyo Medical and Dental University Graduate School of Medicine, Tokyo, Japan

**Keywords:** Influenza, Encephalopathy, Encephalitis, Steroids, Glucocorticoids, Pediatrics

## Abstract

**Background:**

Influenza virus-associated encephalopathy (IAE) can lead to neurological sequela and mortality among children. Therefore, instant recognition and therapeutic intervention for IAE are crucial. In some clinical subtypes of IAE, steroid pulse therapy might be beneficial, especially when it is administered in the early phase. However, early identification of patients who may benefit from steroid pulse therapy is sometimes difficult. We aimed to assess the effectiveness of early steroid pulse therapy among children with IAE.

**Methods:**

In this retrospective observational study, we used a national database that covers half of the acute care inpatients across Japan to identify inpatients aged ≤ 18 years with a diagnosis of IAE between July 2010 and March 2017. Unfavorable outcome was defined as a composite outcome of sequela including Japan Coma Scale ≥ 10 at discharge, requiring tracheostomy, mechanical ventilation, enteral tube feeding, rehabilitation at discharge, or in-hospital death. Propensity score matching was performed to compare unfavorable outcome and in-hospital mortality between patients with and without steroid pulse therapy within 2 days of admission.

**Results:**

Among 692 patients included in the study, the mean age was 5.8 years, and 55.8% were male. The overall in-hospital mortality was 1.3%, and the proportion of the unfavorable outcome was 15.0%. We observed no significant difference in the unfavorable outcome between matched patients (168 patients in each group) with and without early steroid pulse therapy (13.7% vs 8.3%; *P* = 0.16) or in-hospital mortality (0.6% vs 1.2%; *P* = 1.0).

**Conclusions:**

We did not observe the effectiveness of early steroid pulse therapy on patient outcomes among children with IAE in our study population including all clinical subtypes of IAE. Further studies considering severity of illness are warranted to determine whether steroid pulse therapy is beneficial, especially for specific clinical subtypes of IAE.

## Background

Acute encephalopathy, one of the most serious complications among children with influenza virus infection, can lead to neurological sequela and mortality [1]. Neurological symptoms and death occur very early from the onset of influenza symptoms [2]. Instant recognition and intervention for influenza virus-associated encephalopathy (IAE) are therefore crucial.

IAE has been reported worldwide [3–7], but most frequently in Japan [8–10]. This implies difference in genetic background of patients between Japan and other countries [11]. IAE is generally classified according to clinical subtypes including clinically mild encephalitis/encephalopathy with a reversible splenial lesion (MERS), acute encephalopathy with biphasic seizures and late reduced diffusion (AESD), and acute necrotizing encephalopathy (ANE) [8]. In some IAE cases, increased levels of cytokines in the serum or cerebral fluid were observed [12–14], and immunomodulatory therapy such as steroid pulse therapy was therefore proposed and is widely used in addition to systemic support and anti-influenza agents [15–17].

Some previous studies reported that only “early” steroid pulse therapy could be beneficial among children with IAE [15, 17], while most frequent clinical IAE subtypes such as MERS had favorable outcomes without aggressive therapeutic interventions including steroid pulse therapy [8]. However, identification of clinical subtype of IAE or prediction of patient prognosis are sometimes difficult in the early phase when steroid pulse therapy is thought to be beneficial [18, 19]. Therefore, it is necessary to assess whether early steroid pulse therapy is beneficial among children with IAE as a whole.

In this study, we aimed to assess the effectiveness of early steroid pulse therapy on patient outcomes among children with all subtypes of IAE using a nationwide database in Japan.

## Methods

### Study design

This was a retrospective observational study using a nationwide database.

### Database

All data were retrospectively abstracted from the Diagnosis Procedure Combination database in Japan. This database is a national inpatient database that includes administrative claims and discharge abstract data from more than 1000 participating acute care hospitals across Japan. All academic hospitals are obliged to contribute to the database, and community hospitals participate voluntarily. Approximately 50% of all acute-care inpatient data of all ages in Japan are included in the database. The details of this database were described in a previous study [20].

### Participants

Patients aged ≤ 18 years who were admitted to hospitals with a diagnosis of IAE and discharged between July 2010 and March 2017 were included in the study. Diagnosis of IAE was identified according to documented diagnosis of influenza virus infection and encephalopathy during the same hospitalization. Diagnosis of influenza virus infection was determined according to the International Classification of Diseases 10th Revision (ICD-10) codes of J09 (influenza due to identified zoonotic or pandemic influenza virus), J10 (influenza due to identified seasonal influenza virus), and J11 (influenza, virus not identified), as well as a manually documented diagnosis of influenza virus infection. Diagnosis of encephalopathy was determined according to diagnostic codes including virus-associated encephalopathy (ICD-10 code: G948), acute encephalopathy (G934), and delirium due to other medical condition (F058). When a patient was admitted twice or more to the same hospital with a diagnosis of IAE during the study period, only the first admission with a diagnosis of IAE was included.

We excluded the following patients: (1) those transferred from other hospitals because their therapeutic interventions at the initial hospital were unknown, (2) those transferred to other hospitals within 7 days of admission because their outcomes were difficult to determine, (3) those who died within 2 days of admission to avoid immortal time bias, and (4) those aged < 1 month at admission to avoid including unproved congenital diseases such as metabolic disorders.

### Outcomes

Unfavorable outcome was defined as a composite outcome of neurologic sequela and death during the hospital stay including (1) the Japan Coma Scale (JCS) ≥ 10 at discharge, (2) requiring tracheostomy during hospital stay, (3) requiring mechanical ventilation at discharge, (4) requiring enteral tube feeding at discharge, (5) requiring rehabilitation at discharge, and (6) in-hospital death. These unfavorable outcomes did not include those that already existed before admission with IAE.

JCS comprises four categories: alert, 1-digit (awake without stimulation), 2-digit (awake with stimulation), and 3-digit codes (non-awake with stimulation) [21]. Each category has subcategories (1, 2, 3 in the 1-digit code; 10, 20, 30 in the 2-digit code; and 100, 200, 300 in the 3-digit code). JCS 10 indicates that a patient did not open his/her eyes spontaneously; however, he/she opened them in response to a normal volume of voice.

### Therapeutic interventions

Anti-influenza agents included peramivir hydrate, oseltamivir phosphate, laninamivir octanoate hydrate, and zanamivir hydrate. Intravenous antiepileptic drugs included thiopental, thiamylal, propofol, midazolam, diazepam, fosphenytoin, phenytoin, phenobarbital, and levetiracetam. Vasoactive agents included adrenaline, noradrenaline, dopamine, and dobutamine. Steroid pulse therapy was generally administered for 3 consecutive days and comprised 30 mg/kg bodyweight of intravenous methylprednisolone per day [16]. We defined steroid pulse therapy as intravenous administration of any steroid equivalent to methylprednisolone ≥ 30 mg/kg body weight during the hospital stay that was initiated within 2 days of admission.

### Covariates

We collected patient demographic data such as age, sex, body weight at admission, and data on the hospitals they were admitted to. Congenital anomaly was detected according to the ICD-10 codes Q00–99, and epilepsy was detected according to the ICD-10 codes G40 and G41. Epilepsy did not include patients diagnosed after the incidence of IAE. We collected the JCS at admission and at discharge, tracheostomy, mechanical ventilation, enteral tube feeding, and rehabilitation during the hospital stay. Patients with missing data were excluded. We also collected the in-hospital mortality rate of patients, date of admission and discharge, and therapeutic interventions described above.

### Statistical methods

Categorical variables were evaluated using Fisher’s exact test. Continuous variables were evaluated using the Student’s *t* test. Propensity score matching was performed with 1:1 nearest-neighbor matching without replacement using a caliper width of 0.2 of the pooled standard deviation of the logit of the propensity scores between patients with and without steroid pulse therapy [22]. The following factors were included to estimate propensity scores: age category (< 1 year, 1–5 years, 6–11 years, 12–18 years), sex, congenital anomaly, epilepsy at admission, JCS category at admission (0, 1–3, 10–30, and 100–300), and the following factors that were implemented within 2 days of admission: anti-influenza agents, intravenous antiepileptic drugs, acetaminophen, mechanical ventilation, vasoactive agents, computerized tomography or magnetic resonance imaging, cerebrospinal fluid examination, admission to intensive care unit, and immunoglobulin. We calculated absolute standardized differences to check the balance in the distributions of these factors in the unmatched and propensity score-matched groups with and without steroid pulse therapy. Absolute standardized differences of < 10% are generally considered negligible imbalances [23]. We then compared the patient outcomes between the propensity score-matched groups with and without steroid pulse therapy using Fisher’s exact test. A post hoc sensitivity analysis was performed using propensity score matching for patients with an ICD-10 code of G948 (virus-associated encephalopathy) or G934 (acute encephalopathy) and excluding those with code F058 only (delirium due to other medical condition). Statistical significance was defined when two-sided *P* values were < 0.05. All analyses were conducted using Stata version 15.0 (Stata Corp LLC, College Station, Texas).

This study followed the Strengthening the Reporting of Observational Studies in Epidemiology (STROBE) reporting guideline [24] (Supplementary Data).

## Results

### Study population

**Fig. 1:**
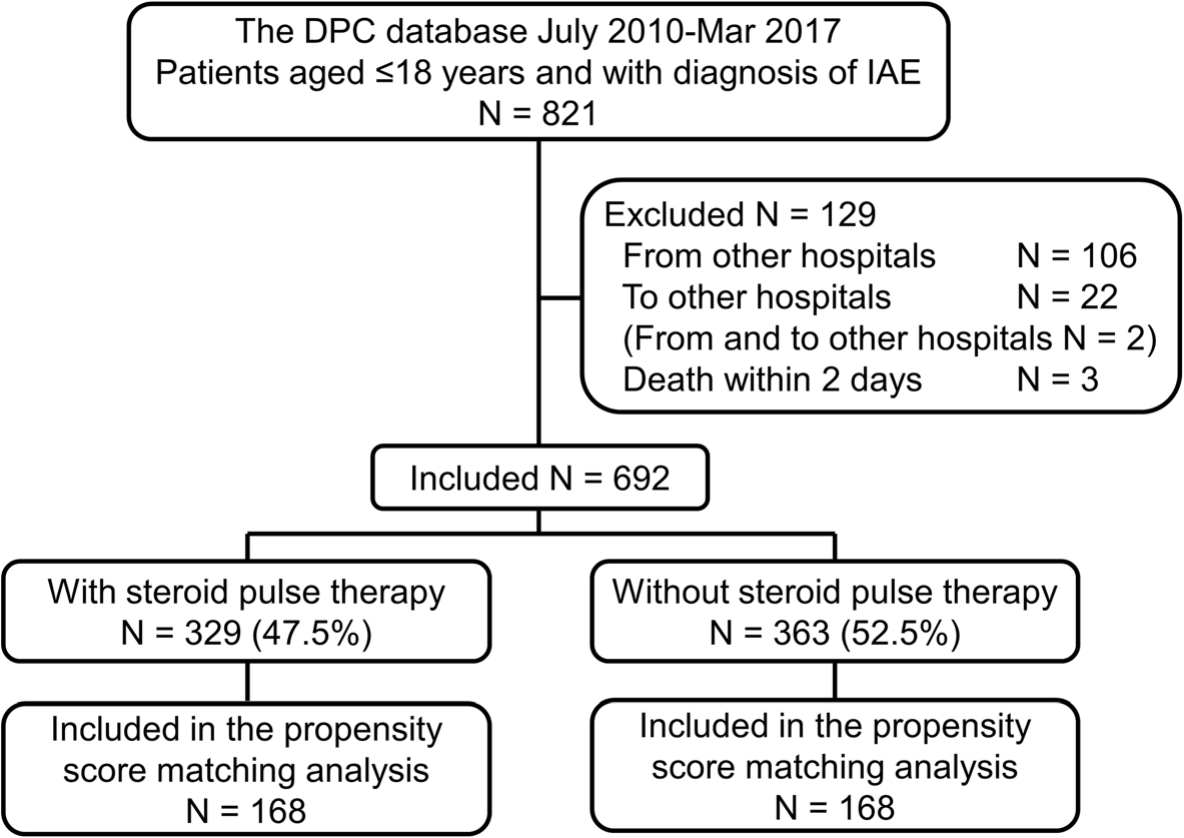
Flowchart of patients in the study. *DPC database* Diagnosis Procedure Combination database, *IAE* influenza virus-associated encephalopathy. On the basis of the International Classification of Diseases 10th Revision, 231 (33.4%) patients out of 692 patients included in the study had the G948 code (virus-associated encephalopathy), 429 (62.0%) had the G934 code (acute encephalopathy), and 45 (6.5%) had the F058 code (delirium due to other medical condition) including duplications. Of these, 11 patients had G948 and G934 codes, 1 had G948 and F058 codes, and 1 had G934 and F058 codes

During the study period, 821 patients aged ≤ 18 years were admitted to hospital with a diagnosis of IAE and discharged. Among these, 106 patients were transferred from other hospitals, 22 patients were transferred to another hospital within 7 days of admission, and 3 patients died within 2 days of admission. Two patients were transferred from other hospitals and were transferred to other hospitals. There was no neonatal patient aged < 1 month. Consequently, 692 patients were included in the analysis (Fig. 1).

Among those included in the analysis, the mean age was 5.8 years (95% confidence interval (CI), 5.5–6.1), 386 (55.8%) patients were male, 8 (1.2%) patients had a congenital anomaly, and 115 (16.6%) patients had preexisting epilepsy at admission. These 692 patients were cared for in 261 hospitals across Japan. The mean length of stay was 13.6 days (95% CI, 11.4–15.8). The overall in-hospital mortality was 15 out of 821 (1.8%) among all children with IAE identified in the database and 9 out of 692 (1.3%) among patients included in the analysis.

### Patient characteristics and therapeutic interventions

Table 1 shows the patient characteristics and therapeutic interventions among unmatched patients with and without early steroid pulse therapy. Among 329 patients with steroid pulse therapy (≥ 30 mg/kg) initiated within 2 days of admission, 235 (71.4%) received ≥ 90 mg/kg of steroids. The median initiation day of steroid pulse therapy was 1.7 days (95% CI, 1.6–1.9). Table 1 also shows the characteristics and therapeutic interventions administered within 2 days of admission between propensity score-matched patients with and without early steroid pulse therapy. Absolute standardized differences were ≤ 10% for most of the characteristics, except for epilepsy (11.6%). There were no missing data in the database regarding the variables included in Table 1.

**Table 1:** Patient characteristics and interventions among unmatched and propensity score-matched patients with and without steroid therapy

	Unmatched patients				Propensity score-matched patients		
	All *N* = 692	With steroid pulse therapy *N* = 329	Without steroid pulse therapy *N* = 363	Absolute standardized difference (%)	With steroid pulse therapy *N* = 168	Without steroid pulse therapy *N* = 168	Absolute standardized difference (%)
Age category							
< 1 year, *n* (%)	27 (3.9)	15 (4.6)	12 (3.3)	6.5	3 (1.8)	4 (2.4)	4.2
1–5 years, *n* (%)	376 (54.3)	189 (57.4)	187 (51.5)	11.9	90 (53.6)	85 (50.6)	6.0
6–11 years, *n* (%)	223 (32.2)	100 (30.4)	123 (33.9)	7.5	57 (33.9)	59 (35.1)	2.5
12–18 years, *n* (%)	66 (9.5)	25 (7.6)	41 (11.3)	12.7	18 (10.7)	20 (11.9)	3.8
Male, *n* (%)	386 (55.8)	174 (52.9)	212 (58.4)	11.1	100 (59.5)	102 (60.7)	2.4
Congenital anomaly, *n* (%)	8 (1.2)	4 (1.2)	4 (1.1)	1.1	1 (0.6)	2 (1.2)	6.3
Epilepsy^a^, *n* (%)	115 (16.6)	67 (20.4)	48 (13.2)	19.2	22 (13.1)	29 (17.3)	11.6
JCS category at admission							
JCS 0, *n* (%)	310 (44.8)	110 (33.4)	200 (55.1)	44.7	73 (43.5)	65 (38.7)	9.7
JCS 1–3, *n* (%)	164 (23.7)	87 (26.4)	77 (21.2)	12.3	40 (23.8)	46 (27.4)	8.2
JCS 10–30, *n* (%)	91 (13.2)	45 (13.7)	46 (12.7)	30.0	26 (15.5)	28 (16.7)	3.2
JCS 100–300, *n* (%)	127 (18.4)	87 (26.4)	40 (11.0)	40.3	29 (17.3)	29 (17.3)	0.0
Anti-influenza agents within 2 days, *n* (%)	493 (71.2)	277 (84.2)	216 (59.5)	57.0	134 (79.8)	132 (78.6)	2.9
Intravenous antiepileptic drugs within 2 days, *n* (%)	384 (55.5)	250 (76.0)	134 (36.9)	85.6	102 (60.7)	109 (64.9)	8.6
Acetaminophen within 2 days, *n* (%)	298 (43.1)	152 (46.2)	146 (40.2)	12.1	78 (46.4)	82 (48.8)	4.8
Mechanical ventilation within 2 days, *n* (%)	75 (10.8)	60 (18.2)	15 (4.1)	45.8	8 (4.8)	12 (7.1)	10.0
Vasoactive agents within 2 days, *n* (%)	24 (3.5)	19 (5.8)	5 (1.4)	23.8	3 (1.8)	5 (3.0)	7.8
CT or MRI within 2 days, *n* (%)	549 (79.3)	310 (94.2)	239 (65.8)	75.8	156 (92.9)	160 (95.2)	10.0
Cerebrospinal fluid examination within 2 days, *n* (%)	313 (45.2)	198 (60.2)	115 (31.7)	59.6	83 (49.4)	85 (50.6)	2.4
Admission to ICU within 2 days, *n* (%)	76 (11.0)	52 (15.8)	24 (6.6)	29.4	13 (7.7)	17 (10.1)	8.3
Immunoglobulin within 2 days, *n* (%)	103 (14.9)	96 (29.2)	7 (1.9)	81.0	6 (3.6)	7 (4.2)	3.1

*JCS* Japan Coma Scale, *CT* Computerized tomography, *MRI* Magnetic resonance imaging, *ICU* Intensive care unit

^a^Epilepsy patients did not include those diagnosed after the incidence of influenza virus-associated encephalopathy

### Clinical endpoint

Among 692 patients included in the analysis, the number of patients with each unfavorable outcome is shown in Table 2. There were 104 (15.0%) patients with the composite unfavorable outcome. The mean length of stay was 8.2 days (95% CI, 7.7–8.8) and 43.7 days (95% CI, 30.6–56.8) among patients with favorable and unfavorable outcomes, respectively (*P* < 0.001). There were no missing data in the database regarding the outcome variables included in Table 2 except for JCS scores for deceased patients; therefore, none of the patients were excluded from the analyses.

**Table 2:** Number of patients with each unfavorable outcome

Outcomes	Number of patients (%)
Composite unfavorable outcome	104 (15.0)
In-hospital death	9 (1.3)
JCS ≥10 at discharge^a^	21 (3.0)
Tracheostomy during hospital stay	8 (1.2)
Mechanical ventilation at discharge^a^	15 (2.2)
Enteral tube feeding at discharge	21 (3.0)
Rehabilitation at discharge	83 (12.0)

Seventy-eight patients had one unfavorable outcome; twelve patients had two; six patients had three; four patients had four; three patients had five; and one patient had six.

*JCS* Japan Coma Scale

^a^Including deceased patients

### Effectiveness of early steroid pulse therapy

Table 3 shows patient outcomes between propensity score-matched patients with and without early steroid pulse therapy. There was no significant difference in the proportions of the unfavorable outcomes between matched patients with and without early steroid pulse therapy (13.7% vs 8.3%; *P* = 0.16) or in-hospital mortality (0.6% vs 1.2%; *P* = 1.0).

**Table 3:** Patient outcomes between propensity score-matched patients with and without steroid pulse therapy

	With steroid pulse therapy *N* = 168	Without steroid pulse therapy *N* = 168	*P* value
Unfavorable outcome ^a^, *n* (%)	23 (13.7)	14 (8.3)	0.16
In-hospital mortality, *n* (%)	1 (0.6)	2 (1.2)	1.0

^a^ Unfavorable composite outcome included (1) Japan Coma Scale ≥ 10 at discharge, (2) requiring tracheostomy during hospital stay, (3) requiring mechanical ventilation at discharge, (4) requiring enteral tube feeding at discharge, (5) requiring rehabilitation at discharge, and (6) in-hospital death

When performing a post hoc sensitivity analysis by propensity score matching for patients with an ICD-10 code of G948 (virus-associated encephalopathy) or G934 (acute encephalopathy) (*N* = 649), the proportions of the composite unfavorable outcomes were 26 out of 166 (15.7%) and 17 out of 166 (10.2%) in patients with and without early steroid pulse therapy, respectively (*P* = 0.19). In this sensitivity analysis, in-hospital mortalities were 0 out of 166 (0%) and 2 out of 166 (1.2%) in patients with and without early steroid pulse therapy, respectively (*P* = 0.50).

## Discussion

In this retrospective observational study, we identified 821 pediatric patients admitted to hospital with a diagnosis of IAE during seven influenza seasons between 2010 and 2017. Of these, we included 692 eligible patients in the analysis and identified that the mortality rate was 1.3%, and the unfavorable rate was 15.0%. We observed no significant difference in patient outcomes between the propensity score-matched patients with and without early steroid pulse therapy in our study population, including all clinical subtypes of IAE.

IAE is generally diagnosed when a patient with influenza virus infection develops neurological symptoms such as delirious behavior, seizure, vomiting, consciousness disorder, and coma [10, 25]. These neurological symptoms usually occur within a few days of influenza virus infection symptoms such as fever, rhinitis, and cough [2]. Although, there are no established diagnostic criteria, we included patients who were clinically diagnosed with IAE by physicians at each hospital and identified those patients according to ICD-10 codes and documented diagnosis in the database.

In our study population, the mean and median ages were 5.8 years and 4.5 years, respectively (the most frequent age was 1 year), and 55.8% were male, similar to previous studies [8, 25]. Additionally, a previous study reported that 84.5% of children with IAE had no underlying disease before the onset of IAE [25], which was similar to our study (82.7% without congenital anomaly or epilepsy). Therefore, most of the patients with IAE were previously healthy children.

Among IAE as a whole, in-hospital mortality was as high as 31.8% in a questionnaire study from 1998 to 1999 [25]; thereafter, it improved to 6.8% in another questionnaire study between 2007 and 2010 [8], was 7.8% in a national surveillance database study between 2010 and 2015 [10], and was 1.8% in our study between 2010 and 2017. Similarly, the proportions of patients with neurologic sequela or mortality were reported as 59.5% in 1998–1999 [25], which improved to 23.6% in 2007–2010 [8] and 15.0% in 2010–2017 in our study population. The differences in mortality and proportions of unfavorable outcomes may be explained by recent improvements in patient outcome among children with IAE [8], differences in case definition and severity of IAE, reporting bias according to the study design, and differences in the definition of unfavorable outcome. However, although patient outcomes including sequela and mortality have improved recently, they remain unacceptable.

IAE includes variety of clinical manifestations such as AESD, ANE, and MERS [1, 8]. In patients with IAE, brain dysfunctions are thought to be caused by cerebral edema [1]. Although the pathogenesis of IAE is poorly understood, it may involve viral invasion to the central nervous system, inflammatory cytokines, metabolic disorders, and genetic susceptibility [26]. Influenza virus is rarely detected in blood or cerebrospinal fluid samples, which suggests direct invasion of the virus into the central nervous system, although this is uncommon [27]. However, increased cytokine levels in serum or cerebral fluid were observed among children with IAE [12–14]. Although chemokines and proinflammatory cytokines play essential defense roles against influenza virus [28], exuberant immune responses with excessive levels of them described as a “cytokine storm“ may lead to extra pulmonary complications including encephalopathy [29]. Therefore, in some clinical subtypes of IAE, immunomodulatory therapies to mitigate the systemic cytokine storm have been proposed. These therapies include steroid therapy [15–17], immunoglobulin [16, 17], cyclosporine [16], and plasma exchange [16]. Among these therapies, steroid therapy in the form of methylprednisolone steroid pulse therapy is documented in the Japanese clinical guidelines for IAE [16] and widely used in Japan [15]. However, such an immunosuppression therapy in patient with infections might lead to more virulent illness, and evidence for these treatments are not sufficient [1]. Therefore, we aimed to assess the effectiveness of steroid pulse therapy among children with IAE using a nationwide database in Japan.

In our study population including all clinical subtypes of IAE, after adjusting for the factors in Table 1, we did not observe a significant difference in the proportions of unfavorable outcomes or in-hospital mortality between the patients with and without early steroid pulse therapy within 2 days. The effectiveness of steroid pulse therapy among children with IAE has not been assessed previously in randomized controlled studies. A previous descriptive study identified the wide use of steroid pulse therapy (39.2%) for IAE in Japan [15]. They observed lower incidences of severe disability or death among children who received “earlier” steroid pulse therapy: 0 out of 9 patients who received steroid pulse therapy within 1 day of the onset of IAE had severe disability or death, 5 out of 11 patients who received therapy on the second day had severe disability or death, and 7 out of 9 patients who received therapy on or after the third day had severe disability or death. However, as described by the authors, the severity of illness at admission was not adjusted for in their study. Another previous study investigated patients with ANE without brain stem lesions (*N* = 17; influenza related, *N* = 5), and observed a lower incidence of unfavorable outcomes among patients who received early steroids: 5 out of 12 patients who received early steroids (within 24 h) had unfavorable outcomes, and 5 out of 5 patients who received steroids after 24 h or who did not receive steroids had unfavorable outcomes (*P* = 0.04) [17]. However, that study did not adjust for the severity of illness at admission, and no difference was observed between patients who received steroids and patients who did not receive any steroids. In our study population including all clinical subtypes of IAE, after adjusting for several factors such as patient characteristics, status at presentation, and therapeutic interventions, we did not observe effectiveness of early steroid pulse therapy. However, patients with some clinical subtypes of IAE may have benefit from the steroid pulse therapy [16, 17]. Therefore, further studies are needed in this regard.

The present study had some limitations. First, this was a retrospective study, and all the data relied on past records in the database. Additionally, we only assessed the Japanese database; therefore, the generalizability of the results is limited. Second, we excluded patients who were transferred from and to other hospitals. Third, data on laboratory results such as higher creatinine [18, 19], lactate dehydrogenase [18, 30], serum aspartate aminotransferase, alanine aminotransferase [19, 30], and lower platelet counts [18] were not available, although they may have been associated with patient outcomes. Similarly, we did not include results for electroencephalography or brain imaging such as computerized tomography and magnetic resonance imaging, which may have influenced the initiation for steroid pulse therapy. Fourth, IAE has several subtypes, which have different prognoses including AESD, ANE, and MERS [8] for which information was not available in the database. Nonetheless, we believe our results are important because identification of specific clinical subtype of IAE is sometimes difficult, especially in the early phase when steroid pulse therapy is thought to be beneficial [18, 19]. Furthermore, although the ICD-10 code of F058 (delirium due to other medical condition) may have included mild IAE cases who did not benefit from the early steroid pulse therapy, the sensitivity analysis that excluded patients with the F058 code showed the same results. Fifth, although we adjusted for potential risk factors such as requiring mechanical ventilation, vasoactive agents, or admission to an intensive care unit, we did not include validated risk adjustment tools in our analyses. Sixth, our composite outcome was not validated. Although validated outcome measures such as Pediatric Cerebral Performance Category (PCPC) scale [31] may be warranted, they were not available in the database. Nonetheless, because the PCPC scale includes patient consciousness or coma status, we included JCS scores, which are thought to be correlated with the Glasgow Coma Scale scores, in our composite outcome [32]. Seventh, we did not identify long-term patient outcomes. A previous study reported that moderate or severe disability was maintained; however, mild disability may have improved thereafter [33].

## Conclusions

In our retrospective observational study, we did not observe a significant difference in the proportions of unfavorable outcomes or in-hospital mortality between the patients with and without early steroid pulse therapy within 2 days of admission among children with IAE as a whole. However, further studies considering severity of illness are warranted to determine whether steroid pulse therapy is beneficial especially for specific clinical subtypes of IAE.

## Supplementary information

**Additional file 1.**

## Data Availability

The datasets generated and analyzed during the current study are not publicly available complying with data-sharing policies of the database.

## Abbreviations

IAE: Influenza virus-associated encephalopathy
MERS: Clinically mild encephalitis/encephalopathy with a reversible splenial lesion
AESD: Acute encephalopathy with biphasic seizures and late reduced diffusion
ANE: Acute necrotizing encephalopathy
ICD-10: International classification of diseases 10th revision
JCS: Japan coma scale
CI: Confidence interval
