# Supplementary material for: Early steroid pulse therapy among children with influenza virus-associated encephalopathy
Source: J Intensive Care. 2020 Aug 12;8:62. doi: 10.1186/s40560-020-00479-8 (PMC7422675; doi:10.1186/s40560-020-00479-8)
Supplement: Supplementary file 1 — Additional file 1. [file 40560_2020_479_MOESM1_ESM.docx]

**STROBE Statement—checklist of items that should be included in reports of observational studies**

|  | Item No. | Recommendation | Page  No. | Relevant text from manuscript |
| --- | --- | --- | --- | --- |
| **Title and abstract** | 1 | (*a*) Indicate the study’s design with a commonly used term in the title or the abstract | P1 | Retrospective observational study |
|  |  | (*b*) Provide in the abstract an informative and balanced summary of what was done and what was found | P1 | We did not observe the effectiveness of early steroid pulse therapy on patient outcomes among children with IAE in our study population including all clinical subtypes of IAE. |
| Introduction | | | |  |
| Background/rationale | 2 | Explain the scientific background and rationale for the investigation being reported | P2 | However, identification of clinical subtype of IAE or prediction of patient prognosis are sometimes difficult in the early phase when steroid pulse therapy is thought to be beneficial. Therefore, it is necessary to assess whether early steroid pulse therapy is beneficial among children with IAE as a whole. |
| Objectives | 3 | State specific objectives, including any prespecified hypotheses | P2 | In this study, we aimed to assess the effectiveness of early steroid pulse therapy on patient outcomes among children with all subtypes of IAE using a nationwide database in Japan. |
| Methods | | | |  |
| Study design | 4 | Present key elements of study design early in the paper | P2 | This was a retrospective observational study using a nationwide database. |
| Setting | 5 | Describe the setting, locations, and relevant dates, including periods of recruitment, exposure, follow-up, and data collection | P2–3 |  |
| Participants | 6 | (*a*) *Cohort study*—Give the eligibility criteria, and the sources and methods of selection of participants. Describe methods of follow-up  *Case-control study*—Give the eligibility criteria, and the sources and methods of case ascertainment and control selection. Give the rationale for the choice of cases and controls  *Cross-sectional study*—Give the eligibility criteria, and the sources and methods of selection of participants | P2 | Study participants were patients aged ≤18 years who were admitted to hospitals with a diagnosis of IAE and discharged between July 2010 and March 2017. |
|  |  | (*b*) *Cohort study*—For matched studies, give matching criteria and number of exposed and unexposed  *Case-control study*—For matched studies, give matching criteria and the number of controls per case | P3, Table 1 | Propensity score matching was performed with 1:1 nearest-neighbor matching without replacement using a caliper width of 0.2 of the pooled standard deviation of the logit of the propensity scores between patients with and without steroid pulse therapy. |
| Variables | 7 | Clearly define all outcomes, exposures, predictors, potential confounders, and effect modifiers. Give diagnostic criteria, if applicable | P2–3 | Outcomes  Therapeutic interventions  Covariates |
| Data sources/ measurement | 8* | For each variable of interest, give sources of data and details of methods of assessment (measurement). Describe comparability of assessment methods if there is more than one group | P2–3 | Outcomes  Therapeutic interventions  Covariates |
| Bias | 9 | Describe any efforts to address potential sources of bias | P2–3 | Outcomes  Therapeutic interventions  Covariates |
| Study size | 10 | Explain how the study size was arrived at | P2, Figure 1 | Participants |

Continued on next page

| Quantitative variables | 11 | Explain how quantitative variables were handled in the analyses. If applicable, describe which groupings were chosen and why | P3 | Categorical variables were evaluated using Fisher’s exact test. Continuous variables were evaluated using the Student’s *t*-test. |
| --- | --- | --- | --- | --- |
| Statistical methods | 12 | (*a*) Describe all statistical methods, including those used to control for confounding | P3 | Propensity score matching was performed with 1:1 nearest-neighbor matching without replacement using a caliper width of 0.2 of the pooled standard deviation of the logit of the propensity scores between patients with and without steroid pulse therapy |
|  |  | (*b*) Describe any methods used to examine subgroups and interactions | P3 | Absolute standardized differences of <10% are generally considered negligible imbalances. Then we compared the patient outcomes between the propensity score matched groups with and without steroid pulse therapy using Fisher’s exact test. |
|  |  | (*c*) Explain how missing data were addressed | P3 | Patients with missing data were excluded. |
|  |  | (*d*) *Cohort study*—If applicable, explain how loss to follow-up was addressed  *Case-control study*—If applicable, explain how matching of cases and controls was addressed  *Cross-sectional study*—If applicable, describe analytical methods taking account of sampling strategy | P2 | Participants |
|  |  | (*e*) Describe any sensitivity analyses | P3 | A post-hoc sensitivity analysis was performed using propensity score matching for patients with an ICD-10 code of G948 (virus-associated encephalopathy) or G934 (acute encephalopathy) and excluding those with code F058 only (delirium due to other medical condition). |
| Results | | | | |
| Participants | 13* | (a) Report numbers of individuals at each stage of study—eg numbers potentially eligible, examined for eligibility, confirmed eligible, included in the study, completing follow-up, and analysed | P3, Figure 1 | During the study period, 821 patients aged ≤18 years were admitted to hospital with a diagnosis of IAE and discharged. |
|  |  | (b) Give reasons for non-participation at each stage | P3, Figure 1 | Among these, 106 patients were transferred from other hospitals, 22 patients were transferred to another hospital within 7 days of admission, and 3 patients died within 2 days of admission. Two patients were transferred from other hospitals and were transferred to other hospitals. There was no neonatal patient aged <1 month. |
|  |  | (c) Consider use of a flow diagram | Figure 1 |  |
| Descriptive data | 14* | (a) Give characteristics of study participants (eg demographic, clinical, social) and information on exposures and potential confounders | P3, Table 1 | Patient characteristics and therapeutic interventions |
|  |  | (b) Indicate number of participants with missing data for each variable of interest | P3–4 | There were no missing data in the database regarding the variables included in Table 1.  There were no missing data in the database regarding the outcome variables included in Table 2 except for JCS scores for deceased patients; therefore, none of the patients were excluded from the analyses. |
|  |  | (c) *Cohort study*—Summarise follow-up time (eg, average and total amount) | P4 | The mean length of stay was 8.2 days (95% CI, 7.7–8.8) and 43.7 days (95% CI, 30.6–56.8) among patients with favorable and unfavorable outcomes, respectively (P < 0.001). |
| Outcome data | 15* | *Cohort study*—Report numbers of outcome events or summary measures over time | P4, Table 2 | Among 692 patients included in the analysis, the number of patients with each unfavorable outcome is shown in Table 2. There were 104 (15.0%) patients with the composite unfavorable outcome. |
|  |  | *Case-control study—*Report numbers in each exposure category, or summary measures of exposure |  |  |
|  |  | *Cross-sectional study—*Report numbers of outcome events or summary measures |  |  |
| Main results | 16 | (*a*) Give unadjusted estimates and, if applicable, confounder-adjusted estimates and their precision (eg, 95% confidence interval). Make clear which confounders were adjusted for and why they were included | P3–4,  Table 1 |  |
|  |  | (*b*) Report category boundaries when continuous variables were categorized | Table 1 | Age category |
|  |  | (*c*) If relevant, consider translating estimates of relative risk into absolute risk for a meaningful time period | Not applicable. |  |

Continued on next page

| Other analyses | 17 | Report other analyses done—eg analyses of subgroups and interactions, and sensitivity analyses | P4 |  |
| --- | --- | --- | --- | --- |
| Discussion | | | | |
| Key results | 18 | Summarise key results with reference to study objectives | P4 |  |
| Limitations | 19 | Discuss limitations of the study, taking into account sources of potential bias or imprecision. Discuss both direction and magnitude of any potential bias | P6–7 |  |
| Interpretation | 20 | Give a cautious overall interpretation of results considering objectives, limitations, multiplicity of analyses, results from similar studies, and other relevant evidence | P4–7 |  |
| Generalisability | 21 | Discuss the generalisability (external validity) of the study results | P6–7 |  |
| Other information | |  | | |
| Funding | 22 | Give the source of funding and the role of the funders for the present study and, if applicable, for the original study on which the present article is based | P7 | Funding |

*Give information separately for cases and controls in case-control studies and, if applicable, for exposed and unexposed groups in cohort and cross-sectional studies.

**Note:** An Explanation and Elaboration article discusses each checklist item and gives methodological background and published examples of transparent reporting. The STROBE checklist is best used in conjunction with this article (freely available on the Web sites of PLoS Medicine at http://www.plosmedicine.org/, Annals of Internal Medicine at http://www.annals.org/, and Epidemiology at http://www.epidem.com/). Information on the STROBE Initiative is available at www.strobe-statement.org.
